# RVFV Infection in Goats by Different Routes of Inoculation

**DOI:** 10.3390/v10120709

**Published:** 2018-12-12

**Authors:** Andrea L. Kroeker, Valerie Smid, Carissa Embury-Hyatt, Estella Moffat, Brad Collignon, Oliver Lung, Robbin Lindsay, Hana Weingartl

**Affiliations:** 1 Canadian Food Inspection Agency, Winnipeg, MB R3E 3M4, Canada; valerie.smid@canada.ca (V.S.); Carissa.emburyhyatt@canada.ca (C.E.-H.); estella.moffat@canada.ca (E.M.); brad.collignon@canada.ca (B.C.); oliver.lung@canada.ca (O.L.); 2 Department of Biological Sciences, University of Manitoba, Winnipeg, MB R3T 2N2, Canada; 3 Public Health Agency of Canada, Winnipeg, MB R3E 3M4, Canada; robbin.lindsay@canada.ca; 4 Department of Entomology, University of Manitoba, Winnipeg, MB R3T 2N2, Canada; 5 Department of Medical Microbiology, University of Manitoba, Winnipeg, MB R3E 0J9, Canada

**Keywords:** Rift Valley fever virus, arbovirus, caprine, challenge model, animal vaccine, zoonosis

## Abstract

Rift Valley fever virus (RVFV) is a zoonotic arbovirus of the Phenuiviridae family. Infection causes abortions in pregnant animals, high mortality in neonate animals, and mild to severe symptoms in both people and animals. There is currently an ongoing effort to produce safe and efficacious veterinary vaccines against RVFV in livestock to protect against both primary infection in animals and zoonotic infections in people. To test the efficacy of these vaccines, it is essential to have a reliable challenge model in relevant target species, including ruminants. We evaluated two goat breeds (Nubian and LaMancha), three routes of inoculation (intranasal, mosquito-primed subcutaneous, and subcutaneous) using an infectious dose of 10^7^ pfu/mL, a virus strain from the 2006–2007 Kenyan/Sudan outbreak and compared the effect of using virus stocks produced in either mammalian or mosquito cells. Our results demonstrated that the highest and longest viremia titers were achieved in Nubian goats. The Nubian breed was also efficient at producing clinical signs, consistent viremia (peak viremia: 1.2 × 10^3^–1.0 × 10^5^ pfu/mL serum), nasal and oral shedding of viral RNA (1.5 × 10^1^–8 × 10^6^ genome copies/swab), a systemic infection of tissues, and robust antibody responses regardless of the inoculation route. The Nubian goat breed and a needle-free intranasal inoculation technique could both be utilized in future vaccine and challenge studies. These studies are important for preventing the spread and outbreak of zoonotic viruses like RVFV and are supported by the Canadian-led BSL4ZNet network.

## 1. Introduction

Rift Valley fever virus (RVFV) is a zoonotic mosquito-borne virus that causes acute infections in ruminants such as goats, cattle, sheep, and camels. Large outbreaks of RVFV have mainly occurred in Sub-Saharan Africa. However, an outbreak outside of the African continent, such as in the Arabian Peninsula in 2001, an imported case in China [1], serological evidence in Turkey [2] and climate changes [3] have raised concerns about the potential spread of the virus to Europe, Asia, and the Americas [4,5,6,7].

RVFV outbreaks in livestock are thought to be primarily transmitted by infected mosquitos. Studies have shown that RVFV epidemics typically follow periods of heavy rainfall conducive to large mosquito populations [8,9,10,11] as well as the trade and importation of infected animals into susceptible regions [12]. In Africa, RVFV is mainly transmitted by the *Aedes aegypti* mosquito, however, a growing number of studies have made it clear that other mosquito species present in Asia, Europe and North/South America are experimentally competent as RVFV vectors [13,14,15]. Therefore, if RVFV were to be introduced into other continents and their endemic mosquito populations, it could cause widespread epidemics and could seriously impact the health of human populations and economically important livestock herds.

RVFV infections in livestock are characterized by abortion storms in pregnant ruminants and high rates of mortality in young sheep, goats, cattle, and camels [16,17]. In contrast, human infections are thought to occur either through mosquito bites or via aerosol transmission from the blood of infected animals [17,18]. For example, the 1977–1978 outbreak in Egypt was identified as a mosquito-transmitted outbreak while the 2009–2011 outbreak in South Africa was primarily an aerosol-transmitted epidemic from exposure to the blood of ruminants during slaughter [18,19,20]; not surprisingly, veterinarians and livestock workers who were in close contact with infected animals were identified as most at risk [21]. Human infection can result in subclinical to severe illness that in some cases can progress to retinal vasculitis, resulting in blindness, encephalitis, and fatal hepatitis with hemorrhagic fever [22]. While reported human case fatality rates are generally low, higher fatality rates (20–40%) were observed in the Kenyan outbreak of 2007–2008 [23] and in Mauritania in 2012 [24]. Therefore, vaccination of livestock against RVFV is an important consideration for both livestock and their associated workers.

There is currently an ongoing effort to produce safe and efficacious vaccines against RVFV in livestock [25,26,27,28,29,30,31,32] as well as reliable challenge models for testing these vaccines. Several groups have developed challenge models for RVFV in cattle [33] and sheep [34,35], and we recently published a challenge model in four month old goats [35,36]. These initial studies have identified important factors of pathogenesis such as infectious dose and the use of insect-derived virus compared to mammalian-derived virus [35]. In addition, several interesting studies have also demonstrated that mosquito saliva can modulate RVFV infection in mice [37]. Interestingly, different routes of transmission have also been studied; for example, aerosol exposure to RVFV led to different disease kinetics and outcomes in mice [38] and non-human primates [39,40] and sheep have been documented to become infected through direct contact [41]. Furthermore, RVFV has been shown to be highly dependent on the viral strain, animal species, breed, and age. Therefore, our goal in this study was to explore how some these factors affected the pathogenicity of our goat model and whether they could be utilized in future vaccine efficacy trials.

## 2. Materials & Methods

### 2.1. Ethics Statement

All animal experiments were carried out in the enhanced biosafety level 3 (BSL3) facility at the National Centre for Foreign Animal Disease (NCFAD) in Winnipeg, Manitoba. All protocols for animal use were approved on 1 March 2017 under the animal use document number C-17-002 at the Canadian Science Centre for Human and Animal Health (CSCHAH) in Winnipeg, Manitoba by the Animal Care Committee. Care was taken to minimize animal suffering and to follow the Canadian Council on Animal Care guidelines for animal manipulations.

### 2.2. Cells

Mosquito C6/36 cells (ATCC, Manassas, VA, USA) were grown and infected in 1:1 EMEM and ESF-921 (Expression Systems, Davis, CA USA) supplemented with 10% fetal bovine serum (FBS) (Hyclone/Fisher Scientific, Ottawa, ON, Canada) and 1% l-glutamine and maintained at 28 °C without CO_2_. Mammalian Vero E6 (VE6) (ATCC) cells were grown and infected in DMEM (Gibco/Fisher Scientific, Ottawa, ON, Canada) supplemented with 10% FBS and maintained at 37 °C with 95% relative humidity and 5% CO_2_.

### 2.3. Virus Production and Titration

A virus isolate from the 2006–2007 Kenyan outbreak (Genbank #MH175203, MH175204, MH175205) was blindly passaged in VE6 cells four times until CPE was visible (titer 2 × 10^5^ pfu/mL). Thereafter, virus was alternatively passaged between VE6 and C6/36 cells [42] and the collected virus was titrated on VE6 cells with a plaque assay to determine virus concentration: At passage 5 the virus was amplified in C6/36 cells (1.4 × 10^5^ pfu/mL); at passage 6 the virus was amplified in VE6 cells (2.3 × 10^6^ pfu/mL); at passage 7 the virus amplified in C6/36 cells (1 × 10^7^ pfu/mL); and at passage 8 the virus was amplified in VE6 cells (1.4 × 10^7^ pfu/mL). The goats were then infected using passage 7 C6/36-derived virus or passage 8 VE6-derived virus.

### 2.4. Genome Sequencing

RNA was converted to cDNA using the Superscript IV First Strand Synthesis Module (Invitrogen/Fisher Scientific, Ottawa, ON, Canada) according to the manufacturer’s specifications except for the following modifications: Eight gene specific primers (10 µM; 0.25 µL each) [43] were used to selectively enrich for RVFV RNA, and a total of 10 µL of RNA was added to the reaction. Second strand synthesis was carried out using the NEBNext mRNA Second Strand Synthesis Module (New England Biolabs, Winnipeg, MB, Canada) according to the manufacturer’s specifications. The double-stranded cDNA was purified using the QIAquick PCR Purification Kit (Qiagen, Toronto, ON, Canada) according to the manufacturer’s specifications and eluted in 20 µL of nuclease-free water. A volume of 2 µL of purified double-stranded cDNA from each sample was quantified on the Qubit 3.0 fluorometer (ThermoFisher Scientific, Burlington, ON, Canada) using the dsDNA High Sensitivity Kit (ThermoFisher Scientific). The samples were diluted to 0.2 ng/µL in nuclease-free water and a total of 5 µL of diluted material was used as input to generate sequencing libraries using the Nextera XT Library Preparation Kit (Illumina, San Diego, CA, USA), then pooled with other libraries before sequencing on an Illumina MiSeq platform using a V2 300-cycle (2 × 150 bp reads) cartridge (Illumina) and Micro flow cell (Illumina).

#### 2.4.1. Sanger Sequencing

Sanger sequencing was used to sequence a short GC-rich area located in the intergenic region of the S segment that had no MiSeq reads mapping to the reference for all samples tested. Briefly, samples were amplified by singleplex PCR using the SuperScript III One-Step RT-PCR System with Platinum Taq DNA Polymerase (Invitrogen) and in-house designed primers (Forward: 5′-CTAGAGGACTCCTTTGTTGG-3′, Reverse: 5′-CTTGAAAGCCTTTGGACTTG-3′) to generate a 505 bp amplicon spanning the intergenic region of the S segment. Thermal cycling was performed for 30 min at 47 °C for reverse transcription, 3 min at 94 °C for initial denaturation, followed by 40 cycles of 15 s at 94 °C, 30 s 47 °C, and 1 min at 68 °C and a final extension for 5 min at 68 °C. Amplicons were sequenced using BigDye Terminator v3.1 technology on an ABI 3130xl Genetic Analyzer system (Applied Biosystems/Fisher Scientific, Ottawa, ON, Canada).

#### 2.4.2. Bioinformatic Sequencing Analysis

Read quality of data from the MiSeq was first visualized in FastQC (v0.11.5) (omicX, Le Petit-Quevilly, France), followed by quality filtering and trimming using Trimmomatic (v0.36 with headcrop 20 and sliding window 4:20) (RWTH Aachen University, Aachen, Germany) and de novo assembly using SPAdes assembler (v3.11.1 in metagenomic mode with default settings) (St. Petersburg State University, St. Petersburg, Russia). The resulting assembled contigs were then characterized with blastn to determine their closest match within the nr/nt database. The closest full-length sequence match for each of the three viral gene segments was then used to perform a reference assembly with the raw data (Geneious v9.1.5 on Low Sensitivity/Fastest setting) (Biomatters, Auckland, New Zealand). Finally, for each sample MiSeq and Sanger sequencing data were combined to generate a consensus sequence for each segment (Geneious v9.1.5).

#### 2.4.3. Phylogenetic Analysis

The evolutionary history of the RVFV UAP strain was inferred by using the Maximum Likelihood method based on the JTT matrix-based model [44]. The bootstrap consensus tree inferred from 1000 replicates [45] is taken to represent the evolutionary history of the taxa analyzed [45]. Branches corresponding to partitions reproduced in less than 50% bootstrap replicates are collapsed. The percentage of replicate trees in which the associated taxa clustered together in the bootstrap test (1000 replicates) is shown next to the branches [45]. Initial trees for the heuristic search were obtained automatically by applying Neighbor-Join and BioNJ algorithms to a matrix of pairwise distances estimated using a JTT model, and then selecting the topology with superior log likelihood value. The analysis involved 10 amino acid sequences. There were a total of 2128 positions in the final dataset. Evolutionary analyses were conducted in MEGA7 [46].

### 2.5. Goat Inoculation

Twenty-nine healthy 4 month old Nubian or LaMancha goats were obtained from breeders in Manitoba, Canada and were allowed 7 days to acclimatize to BSL3+ containment at NCFAD, during which they were monitored daily for any signs of disease. After acclimatization, the goats were divided into groups of 2–4 and housed in separate cubicles.

#### 2.5.1. Subcutaneous (Nubian and LaMancha Goats)

A group of Nubian and LaMancha goats were inoculated subcutaneously with 10^7^ pfu in 1 mL insect-derived RVFV. A group of controls were also inoculated subcutaneously with PBS.

#### 2.5.2. Mosquito-Primed Subcutaneous (Nubian Goats Only)

A 50 mL Falcon tube was filled with fifty naïve *Aedes aegyti* mosquitos and the open end was covered with a fine mesh. The mesh side was then placed on a shaved surface on the goat’s necks for 20 min so that the mosquitos could feed. This same surface area was then subsequently inoculated subcutaneously with 10^7^ pfu in 1 mL insect-derived RVFV.

#### 2.5.3. Intranasal (Nubian and LaMancha Goats)

One group of Nubian and one group of LaMancha goats were each inoculated intranasally with 10^7^ pfu insect-derived RVFV. A second group of LaMancha goats was inoculated intranasally with 10^7^ pfu mammalian-derived RVFV. Each goat was given a total of 1 mL virus using a 2 mL syringe, with half given in each nostril.

### 2.6. Clinical Scoring, Goat Sampling & Tissue Collection

#### 2.6.1. Scoring

All goats were monitored for clinical symptoms and rectal temperatures daily. We then calculated a clinical score for the group based on the following characteristics: General appearance (normal = 0, mild disease = 1, disease = 2); fever in any animal (temperature < 41 = 0, temperature ≥ 41 = 1); disposition (bright/alert/responsive = 0, quiet/alert/responsive = 1, depressed = 2); eating habits (normal = 0, less than normal = 1, very little/nothing = 2), drinking habits (normal = 0, nothing = 2), and consistency of feces (normal = 0, clumped/soft = 1, diarrhea = 2). Using our scale, a maximum score of 11 was possible and generally included only mild signs of disease; however, not eating or drinking for more than one day was considered an endpoint.

#### 2.6.2. Sampling

Blood for serum isolation and oral and nasal swabs were collected prior to infection and daily for the first 7 days post infection. Blood, oral and nasal swabs were also collected on days 14, 21, and 28 post infection. Serum samples and swabs were stored at −70 °C.

#### 2.6.3. Tissues

Tissues from infected animals were collected at day 1 (*n* = 1), day 7 (*n* = 1) and day 28 (*n* = 2) post infection and tissues from uninfected control goats were all collected on day 28. Tissues were either placed in 10% formalin for downstream tissue sectioning or processed as 10% homogenates in DMEM (Gibco) for RNA extraction and virus isolation.

### 2.7. Virus Isolation by Plaque Assay

Serial dilutions of serum, nasal swabs and oral swabs were used to infect confluent monolayers of VE6 cells for 1 h. The virus was then removed and the cells were overlayed with 1.75% carboxymethylcellulose (CMC, MilliporeSigma, Oakville, ON, Canada). After 3 days the cells were formalin-fixed and stained with 0.5% crystal violet (MilliporeSigma) to visualize and count plaques.

### 2.8. RNA Isolation and RT-PCR

RVFV RNA was extracted from serum using the TriPure Isolation Reagent (Roche, Laval, QC, Canada) according to the manufacturer’s instructions. Purified RNA was stored at −70 °C. We detected viral RNA with a one-tube real-time polymerase chain reaction (RT-PCR) mix (Rotor-Gene Dual Probe kit, Qiagen) [47] as per the manufacturer’s instructions and ran the samples on the Rotor Gene PCR machine with the following conditions: 30 min at 50 °C, 2 min at 95 °C and 45 cycles of 15 s at 95 °C, and 30 s at 60 °C. Primers (Invitrogen) and probe (Biosearch Tech, Petaluma, CA, USA) targeted nucleotides 2912 to 3001 for the RVFV L gene segment [5]. All Ct values were plotted on a standard curve using a DNA plasmid containing the targeted RVFV L gene segment (GenScript, Piscataway, NJ, USA) and quantified.

### 2.9. In Situ Hybridization of RVFV-Probe in Tissues

Five-micron paraffin-embedded formalin fixed tissue sections were cut, air-dried, and melted onto charged slides in a 60 °C oven. The slides were then cleared and hydrated in xylene and 100% ethanol, and then air-dried. The sections were quenched for 10 min in aqueous H_2_O_2_, boiled in target retrieval solution for 15 min, rinsed in 100% ethanol and air-dried again. A final treatment of protease plus enzyme for 15 min at 40 °C was applied. Next, the probe (V-RVFV-ZH501-NP, from Advanced Cell Diagnostics, Newark, CA, USA) was applied and incubated at 40 °C for 2 h. The hybridization amplification steps (AMP 1–6) were applied to the slides for the recommended times and temperatures as per the manual for the RNAscope^®^ 2.5HD Detection Reagent—the Red kit (Advanced Cell Diagnostics). The signal was then visualized with Fast Red after which the slides were counterstained with Gill’s hematoxylin, dried, cleared and cover-slipped.

### 2.10. Neutralizing Antibody Detection (PRNT)

The presence of neutralizing antibodies to RVFV was determined by a plaque reduction neutralization test (PRNT). Serial 2-fold dilutions of serum were prepared in PBS and incubated with an equal volume of insect-derived RVFV for 1 h at room temperature. Thereafter, 75 µL of the sera-virus mixture was adsorbed to confluent monolayers of VeroE6 cells in 48-well plates in triplicate for 1 h at 37 °C, 5% CO_2_, and 95% relative humidity. A carboxymethylcellulose (MilliporeSigma) overlay was then added to all wells and plates were further incubated at 37 °C, 5% CO_2_, and 95% relative humidity. At 4 days post infection, the cells were fixed with 10% formalin, stained with 0.5% crystal violet, and plaques were counted. The reciprocal of the highest serum dilution that reduced plaques by 70% CPE was read as the antibody-PRNT_70_ titre for that sample.

## 3. Results

### 3.1. Phylogenetic Analysis of a 2006–2007 Strain of the Kenya-UAP RVFV Strain

**Figure 1:**
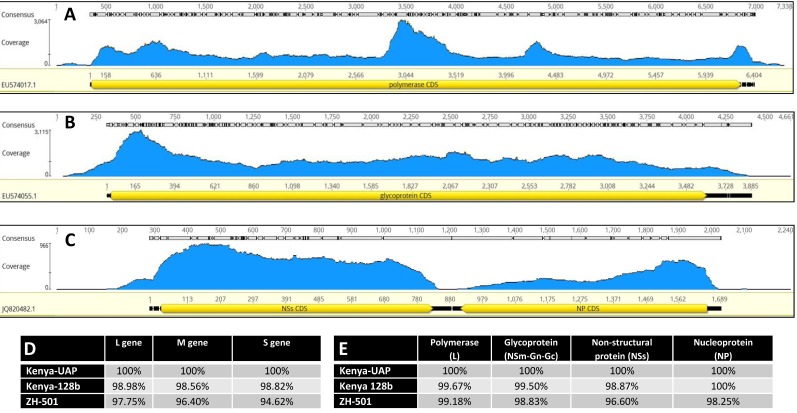
Sequence of RVFV strain UAP. Next generation sequencing was used to sequence the three genome segments of RVFV: L (**A**), M (**B**), and S (**C**). In each graph, the *y*-axis indicates the number of reads and *x*-axis represents the coverage over the length of the gene in base pairs. The nucleotide (**D**) and corresponding amino acid (**E**) sequences of the three RVFV genome segments of UAP were aligned with ZH-501 and Kenya-128-b RVFV strains; results of % homology between the strains are presented for each genome segment.

**Figure 2:**
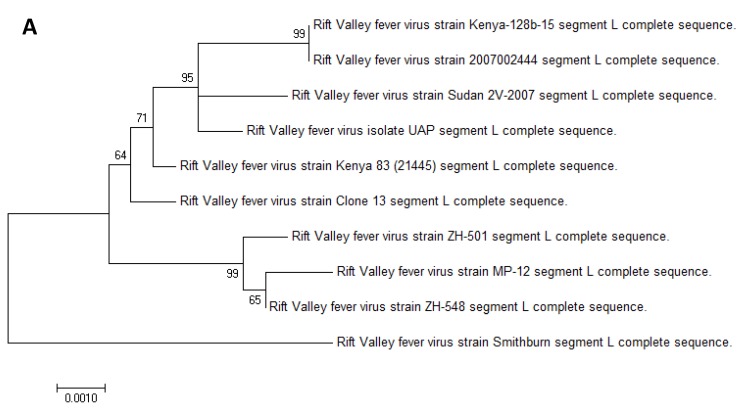

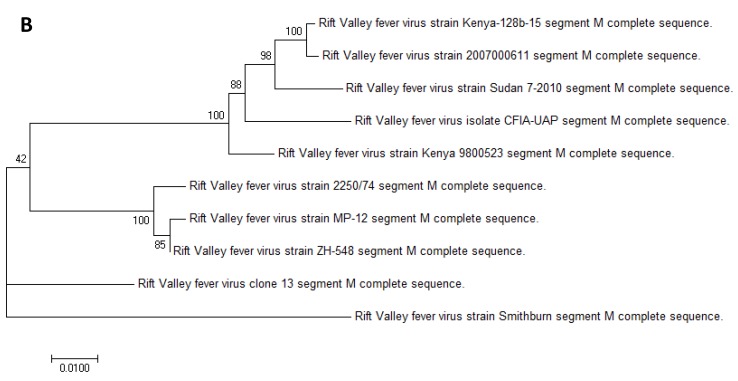

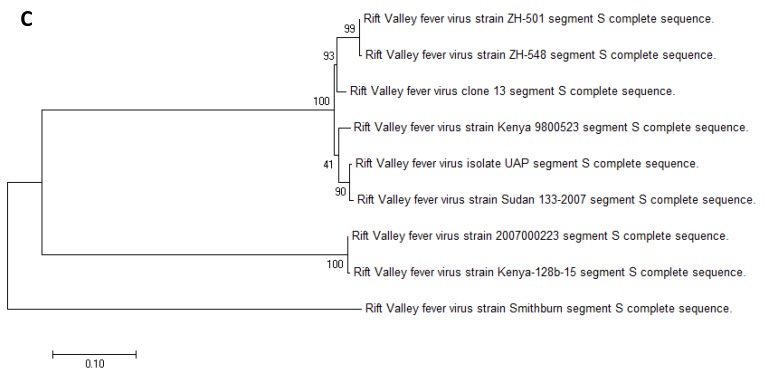
Phylogenetic analysis of RVFV strain UAP A phylogenetic analysis of the RVFV strain UAP using the Maximum-Likelihood Model was performed using MEGA7 software for each of the three RVFV genome segments: L (**A**), M (**B**), and S (**C**).

The RVFV strain used in this study was sequenced and compared to other published RVFV sequences. Greater than 99% coverage of the reference ZH-501 strain and an average read depth of 400–2000 reads per nucleotide was achieved (Figure 1A–C). We also directly compared sequences of our isolate to the Ken-128-b strain used in another study [33,34], as well as the commonly used ZH-501 strain. Our strain matched these with 96.6–99.18% identity at the amino acid level (Figure 1E) and with 94.6–97.75% at the nucleotide level (Figure 1D). We found this sequence homology to be of significance, in that any differences between this study and our previous study with Boer goats and the ZH-501 strain is likely more attributable to the goat breed or the inoculation method than the virus strain. In addition, a phylogenetic comparison demonstrated that the L, M, and S segments all clustered with other strains isolated during the 2006–2007 outbreak from Sudan or Kenya (Figure 2A–C, respectively).

### 3.2. Experimental Design

We compared three different routes of RVFV infection in the Nubian and LaMancha goat breeds (Figure 2A,B). Five groups were infected with virus derived from the C6/36 (C6) mosquito cell line and one group was infected with virus grown in the Vero E6 (VE6) mammalian cell line. The subcutaneous (SC) infection method is widely used in RVFV infection models [33,34,35,39,47] and allows the primary target cells, dermal dendritic cells [48,49], to be infected. Importantly, the subcutaneous group allowed our 2006-2007 RVFV strain to be compared to many other published studies. Our second route of inoculation consisted of a mosquito-primed subcutaneous infection. Several interesting studies in mice have demonstrated that mosquito saliva can modulate RVFV infection [37] as well as other arbovirus infections [50,51,52] and we sought to evaluate this effect in a large animal model. Thirdly, several groups were infected intranasally (IN) as a few studies have shown that aerosol exposure to RVFV could lead to different disease kinetics and outcomes in mice [38] and non-human primates [39,40].

### 3.3. Clinical Signs and Gross Pathology

**Figure 3:**
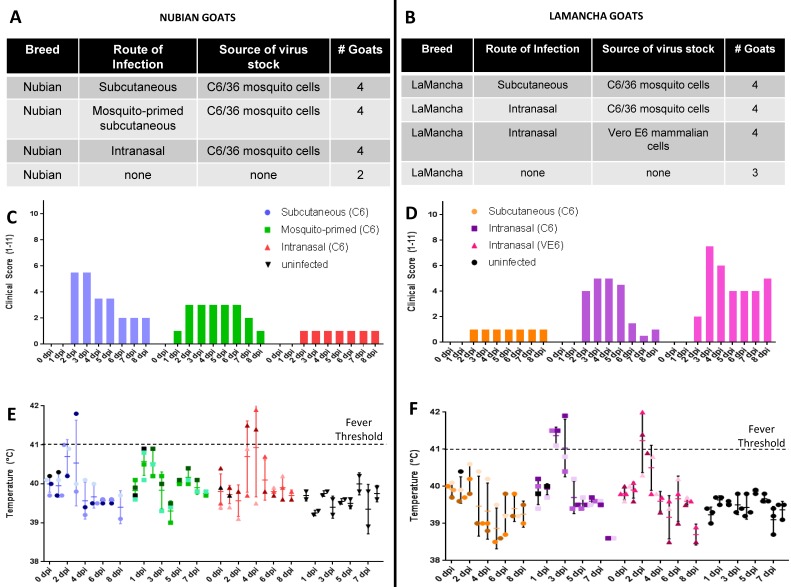
Experimental design and clinical signs. The differences between and number of animals in the experimental groups are summarized for the Nubian (**A**) and LaMancha (**B**) goats. Nubian (**C**) and LaMancha (**D**) goats were examined daily for signs of illness and each experimental group was collectively given a clinical score between 0 and 11. Rectal temperatures were taken and recorded daily for the Nubian (**E**) and LaMancha (**F**) goats. The threshold for fever was considered to be a temperature of 41 °C or higher. Temperature values are shown for individual goats (*n* = 4 at 0–1 dpi; *n* = 3 at 2–7 dpi; and *n* = 2 at 8 dpi); the horizontal line represents the average for the group; the vertical line represents the standard deviation for the group. C6 refers to virus that was grown in C6/36 mosquito cells; VE6 refers to virus that was grown in VE6 mammalian cells. The Nubian and LaMancha uninfected groups received an injection of PBS but no virus.

Inoculation of both the Nubian and LaMancha goats with RVFV resulted in mild clinical signs in all of the groups during the first week of infection. In the Nubian goats, the subcutaneous group reached the highest clinical score (two–five out of 11), the mosquito-primed group was intermediate (one–three out of 11) and the intranasal was the lowest (one out of 11) (Figure 3C). The LaMancha goats had higher clinical scores overall with the intranasal groups reaching the highest scores (two–seven out of 11) and the subcutaneous exhibiting the lowest score (two–five out of 11) (Figure 3D). The extent of observed clinical signs included a mild fever (Nubians Figure 3E; LaMancha Figure 3F), clumped stool, diminished eating, and mild depression.

In the Nubian goats all signs generally resolved by eight-10 dpi. In the LaMancha goats, most clinical signs improved at eight-10 dpi, but did not resolve completely (data not shown). However, ringworm was detected in all LaMancha goats at 14 dpi and likely contributed to the continued presence of mild clinical signs, primarily clumped stool. We postulated that the LaMancha goats had arrived with a latent ringworm infection that may have become apparent during the experiment. Finally, we performed necropsies and collected tissues at one, seven, and 28 dpi. We did not detect any gross pathological changes at these time points.

### 3.4. Viremia, Shedding and Tissue Viral Load

**Figure 4:**
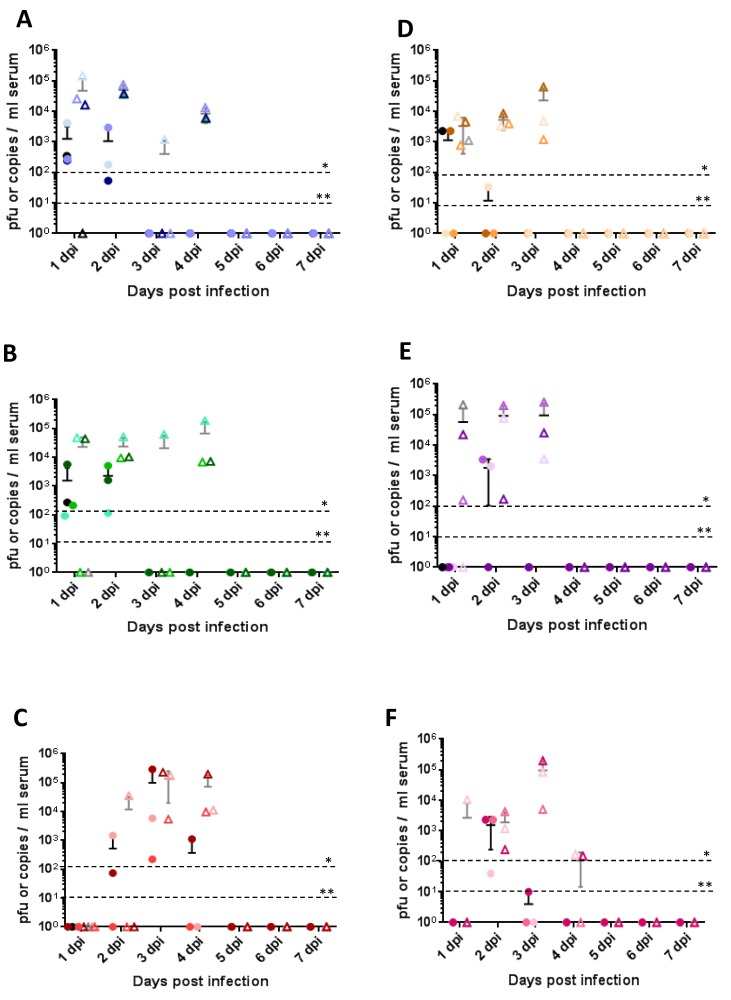
Quantification of viremia. (**A**–**C**) Quantification of RVFV viremia in Nubian goats after subcutaneous infection (**A**), mosquito-primed subcutaneous infection (**B**), and intranasal infection (**C**). (**D**–**F**) Quantification of RVFV viremia in LaMancha goats after subcutaneous infection (**D**), intranasal infection with mosquito cell-derived virus (**E**), and intranasal infection with mammalian cell-derived virus (**F**). Triangles indicate the presence of viral RNA as measured by RT-PCR; circles indicate the presence of infectious virus as measured by plaque assay. Values are shown for individual goats (*n* = 4 on day 1; *n* = 3 on days 2–7); the horizontal line represents the average for the group; the vertical line represents the standard deviation for the group. C6 refers to virus that was grown in C6/36 mosquito cells; VE6 refers to virus that was grown in VE6 mammalian cells. The PCR and plaque detection thresholds are indicated. Dpi: days post infection. * threshold for RNA detection; ** threshold for plaque detection.

All routes of inoculation in both Nubian and LaMancha goats led to infection and consistent viremia, where viremia was detectable for two–four days by RT-PCR (Figure 4A–F, triangles) and for two days by virus isolation (Figure 4A–F, circles). Interestingly, while subcutaneous and mosquito-primed subcutaneous routes led to viremia already on day one post infection, viremia in the intranasal group was delayed until day two or three post infection and correlated with a delayed fever (Figure 3E,F). Clinical signs generally appeared a day after the appearance of viremia and lasted until several days after viremia had been cleared. Clinical scores did not necessarily correlate with the quantity of virus detected though. In the LaMancha goats, a similar trend was seen with the mosquito-derived virus groups; however, viremia tended to be a day shorter than in the Nubian goats (Figure 4D–F). Of all the groups, the highest viremia peak titer reached was 10^5^ pfu/mL in the Nubian intranasal group.

**Figure 5:**
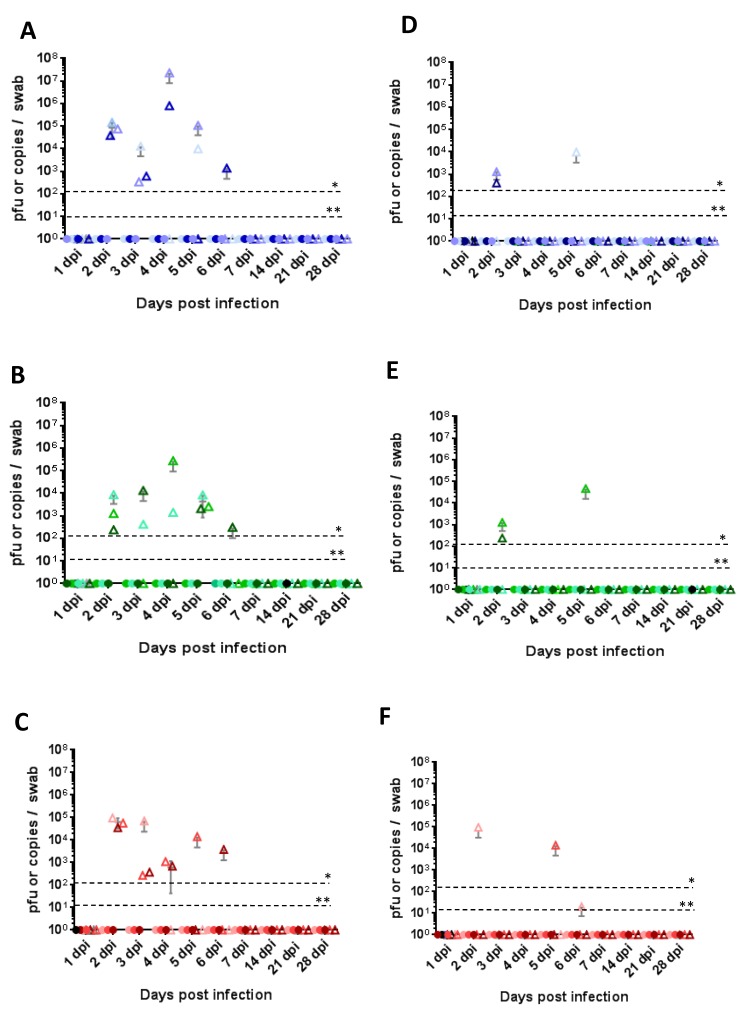
Oral and nasal shedding. (**A**–**C**) Quantification of RVFV in nasal swabs in Nubian goats after subcutaneous infection (**A**), mosquito-primed subcutaneous infection (**B**), and intranasal infection (**C**). (**D**–**F**) Quantification of RVFV in oral swabs in Nubian goats after subcutaneous infection (**D**), mosquito-primed subcutaneous infection (**E**), and intranasal infection (**F**). All LaMancha swabs were negative for virus and data is not shown. Triangles indicate the presence of viral RNA as measured by RT-PCR; circles indicate the presence of infectious virus as measured by plaque assay. Values are shown for individual goats (*n* = 4 on day 1; *n* = 3 on days 2–7; and *n* = 2 on days 14–28); the horizontal line represents the average for the group; the vertical line represents the standard deviation for the group. C6 refers to virus that was grown in C6/36 mosquito cells; VE6 refers to virus that was grown in VE6 mammalian cells. The PCR and plaque detection thresholds are indicated. Dpi: Days post infection. * threshold for RNA detection; ** threshold for plaque detection.

All of the Nubian goats had nasal (Figure 5A–C) and oral (Figure 5D–F) shedding of viral RNA (10^3^–10^7^ copies/swab) at 2–6 dpi; however no infectious virus was detected. In contrast, neither viral RNA nor infectious virus was found in nasal or oral swabs from any of the LaMancha goats (data not shown).

**Figure 6:**
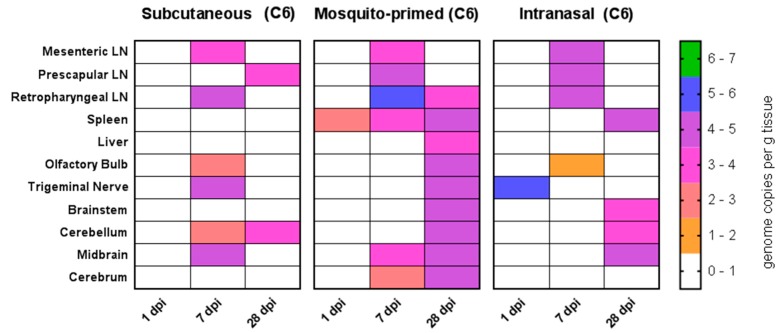
Viral load in tissues. Quantification of RVFV in tissues in Nubian goats at 1 dpi (*n* = 1), 7 dpi (*n* = 1), and 28 dpi (*n* = 2). Only data for the detection of viral RNA by RT-PCR is shown; virus isolations for all tissues were negative. All LaMancha tissues were negative for virus and data is not shown. Dpi: Days post infection. C6 refers to virus that was grown in C6/36 mosquito cells. LN: Lymph node.

In the Nubian goats, all routes of inoculation also led to a systemic spread of the virus into the tissues. While no infectious virus was isolated at our time points, viral RNA was detectable in the spleen, liver, lymph nodes, and a variety of brain tissues (Figure 6). Our first time points consisted of one dpi to catch early localization of the virus in vivo. Indeed, we detected viral RNA in the trigeminal nerve in the intranasally infected group and in the spleen in the mosquito-primed-SQ group (Figure 5A). By seven dpi, the mesenteric, prescapular, and retropharyngeal lymph nodes were generally positive for viral RNA in all groups (Figure 6) (10^2^–10^6^ copies/g tissue) and a few remained positive at 28 dpi (Figure 6). The spleen was positive for the duration of the experiment in the mosquito-primed group (10^2^–10^4^ copies/g tissue), but only appeared by day 28 in the intranasal group (Figure 6) and not at all in the subcutaneous group. In contrast, viral RNA was not detected in the liver except at day 28 for the mosquito-primed group (Figure 6). Virus had begun to invade brain tissue in the subcutaneous and mosquito-primed groups by seven dpi (Figure 6), and in the intranasal group by 28 dpi. Notably, virus was particularly widespread throughout all the brain tissues tested in the mosquito-primed-SC group at 28 dpi (Figure 6). In contrast, neither viral RNA nor infectious virus was found in any tissues in the LaMancha goats at one, seven, or 28 dpi (data not shown).

### 3.5. In-Situ Hybridization for RVFV

**Figure 7:**
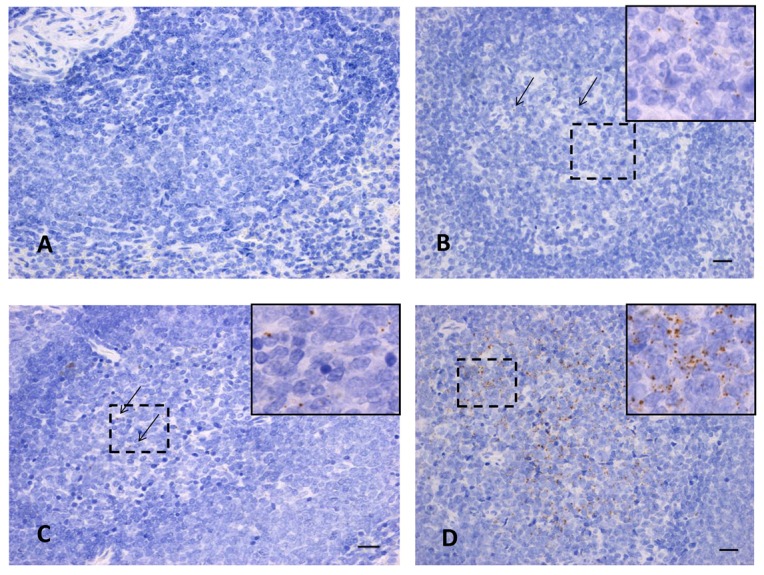
In situ hybridization for RVFV in tissues. In-situ hybridization staining for RVFV in spleen tissues from Nubian goats. (**A**) subcutaneous infection at 7 dpi (*n* = 1), (**B**) subcutaneous infection at 28 dpi (*n* = 2), (**C**) mosquito-primed subcutaneous infection at 28 dpi (*n* = 2), and (**D**) intranasal infection at 28 dpi (*n* = 2). The large images are taken at 40× magnification and arrows indicate individual dots in the slides where weak staining is present. The inserted panels in (**B**–**D**) are taken at 100× magnification to help visualize an area of positive staining, as shown within the dotted lines.

We also confirmed the presence of virus in selected tissues using in-situ hybridization against the RVFV nucleoprotein (NP) sequence. For example, the spleen had positive staining at day 28 in all groups (Figure 7B–D), and at day seven in the mosquito group (data not shown, but it is similar to Figure 7B,C). In contrast, the Nubian subcutaneous and intranasal groups had no staining at day seven and none of the Nubian groups had staining at day one (Figure 7A is a representative image of negative staining in the spleen). Any positive staining was scattered only throughout the spleen follicles in the white pulp and ranged from weak (Figure 7B,C) to strong staining patterns (Figure 7D). Liver samples from the subcutaneous and mosquito-primed groups on day 28 were negative for virus staining. Similarly, the trigeminal nerve was negative for virus straining from the intranasal group on day one, from the subcutaneous group on day 7 and from the mosquito-primed group on day 7.

### 3.6. Neutralizing Antibodies

**Figure 8:**
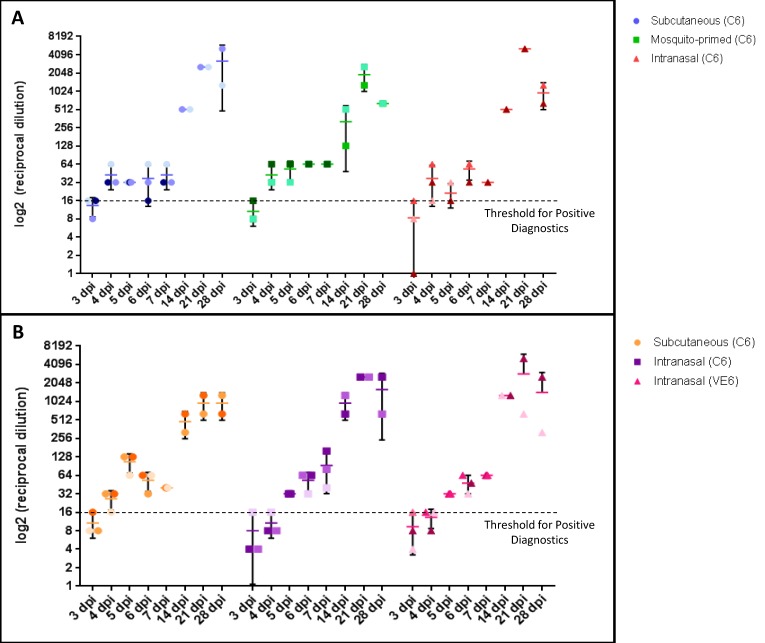
Quantification of neutralizing antibodies against RVFV in serum. The titers are given as a log_2_ reciprocal dilution for the Nubian (**A**) and LaMancha (**B**) goats. Data for individual animals is shown with the horizontal bars representing the standard deviations (*n* = 3 on days 2–7; *n* = 2 on days 14–28). Dpi: Days post infection.

We detected neutralizing antibodies against RVFV in both Nubian (Figure 8A) and LaMancha (Figure 8B) goats starting at four–five days post infection. In both goat breeds, all groups displayed similar titers and kinetics with a peak at 21 days post infection and peak titers of 1/1280 to 1/5120.

## 4. Discussion

Robust challenge models for RVFV are an important prerequisite for testing novel therapeutics and vaccines and need further development for goats. We have previously established the necessary dose required for infection [35] and have demonstrated that mosquito cell-derived virus leads to more consistent viremia compared to the mammalian cell-derived virus [35]. In this follow-up study, we investigated whether other parameters affect the pathogenicity of RVFV in goats including different breeds of goats, routes of inoculation, and a strain of RVFV from the Kenyan/Sudan outbreak in 2006–2007.

Sequencing and phylogenetic analysis of the virus isolate CFIA-Kenya-UAP determined that the virus clusters with other strains primarily isolated from Sudan, and that its origin is distinct from the Kenyan-128b strain utilized by Faburay and Wilson et al. as well as other commonly used strains such as the Egyptian ZH-501 and ZH-548 strains.

Our previous experiments utilized Alpine-Boer goats infected with the RVFV ZH-501 strain. While infection produced consistent viremia and fever, it did not result in observable clinical disease or significant gross pathology [35]. In contrast, we found that the Kenyan strain used in this study induced mild to moderate clinical symptoms in both the Nubian and LaMancha goats that lasted throughout the acute infection phase (days two–five post infection). Notably, this was not likely due to increased infectious virus production as peak and length of viremia in the LaMancha goats were similar to what we saw in the Alpine-Boer goats. Of the three goat breeds tested, Nubians infected intranasally with the Kenyan strain reached the highest peak viremia titers (10^5^ pfu/mL).

Viral spread to the tissues differed greatly between the goat breeds, with the LaMancha completely lacking virus in any tissue tested at any time point, whereas many organs tested positive for viral RNA in the Nubians at both seven and 28 dpi. We first looked at a variety of lymphoid tissues including the spleen that is well known to become infected in sheep and cattle [33,34]. We then investigated other immune tissues including the mesenteric, prescapular, and retropharyngeal lymph nodes. Dendritic cells are thought to be the primary cells to be infected by RVFV, and once activated, they travel to the lymph nodes. We hypothesized that lymph nodes close to the site of infection might be preferentially infected or contain higher amounts of virus. The prescapular lymph node was chosen for its proximity to the subcutaneous injection site behind the shoulder blade, the retropharyngeal lymph node for its proximity to the nasal and oral cavity, and the mesenchymal lymph node as a site distant from either inoculation site. As viremia is present already at one or two dpi, we chose an early time point to look at the lymph nodes. While we did not find detectable virus in the lymph nodes at one dpi and could not distinguish any kinetics, all of the lymph nodes were generally infected in all groups by seven dpi, indicating systemic circulation of the virus.

Previous studies in calves, sheep, and non-human primates have found extensive involvement of the liver in RVFV pathology including lesions, the presence of viral RNA and infectious particles, and changes in blood liver enzymes on days three, four, and five [33,34,40,53]. In contrast, we did not detect high levels of viral RNA in the liver in the Nubian or LaMancha goats by PCR, did not detect viral RNA by in situ hybridization and all liver samples were negative for infectious particles. The only liver sample that was PCR-positive for RVFV was in the Nubian mosquito-primed goats at day 28. With large animals, it is always possible that we simply did not sample an area of the liver that was positive, although we attempted to sample randomly from different areas or would have selected lesions if they had been present. We think it is most likely that the liver was mainly infected during the acute phase of infection, and any liver lesions or impairment may have been resolved in the goats by seven dpi as this is consistent with previous publications [33,34].

Interestingly, we detected substantial levels of viral RNA in different brain tissues in the Nubian goats, although all brain tissues were negative for infectious virus. For example, at one day post infection the trigeminal nerve was positive for viral RNA in the intranasally-infected group. This time point is particularly notable in that it detected neural infection prior to the presence of viremia. This would suggest that neurons could be directly targeted during an intranasal exposure, rather than occurring through viremia and a breakdown of the blood brain barrier. Unfortunately though, we were unable to confirm the presence of virus in the trigeminal nerve with in-situ hybridization. We also detected viral RNA in various brain tissues at seven dpi in the subcutaneous and mosquito-primed Nubian groups and in all groups by 28 dpi. The most consistently infected brain tissues included the cerebellum, midbrain, and brainstem. RVFV has been detected in the brain in a few other studies as well. For example, in a mouse model of aerosolized RVFV the authors found virus in the neuroepithelium of the olfactory bulb at seven days post infection [38]. In addition, the neurons of 21-day old calves at nine days after subcutaneous infection were positive for RVFV [53]. In vitro cultures of brain tissues from a variety of different ruminants have also been shown to support robust RVFV replication [54]. Notably, our study is the first indication of infected brain tissue in goats, the first to show positivity at such an early time point post infection and the only study to have investigated different regions of the brain.

Whether a difference actually existed between the subcutaneous and mosquito-primed groups in the Nubian goats was difficult to conclude. Certainly, there was no difference in viremia between the two groups. However, the subcutaneous group had a greater number of RVFV RNA positive brain tissues at seven dpi (*n* = 1), while the mosquito-primed group had a great number of RVFV RNA positive brain at 28 dpi (*n* = 2). While this is somewhat striking, the numbers of animals are very small and we cannot discern from this study whether these are due to differences in individual animals or due to the infection method.

In ruminants RVFV transmission appears to be primarily mosquito driven, although results from transmission experiments have been mixed. For example, a few studies in sheep have demonstrated transmission through oral and respiratory routes [41,55,56,57] as well as through direct contact [41]. We hypothesized that the intranasal infection group might shed virus, and consequently, we measured viral shedding in both nasal and oral swabs. Surprisingly, all three routes of inoculation induced high and similar levels of shedding of viral RNA throughout acute infection in the Nubian goats, while shedding was completely absent in the LaMancha goats. This highlights a potential utility for nasal swabs in diagnostic testing; however, shedding may be breed specifically and requires further investigations for reliability. In contrast, the oral swabs were not as consistent in detecting viral RNA. We think this is likely partly attributable to the fact that we took oral swabs from goats who were alert and active; it was often difficult to get comprehensive coverage of the oral membranes. However, viral RNA was sporadically detected in oral swabs and indicates that a different sampling method would perhaps produce better results. We did not detect any infectious virus in either oral or nasal swabs, suggesting that under our laboratory conditions the risk of transmission from shedding is very low. It is possible that there are high levels of interfering particles present in these samples, which would lead to a much higher viral RNA load compared to infectious virus. We also considered the possibility that inhibitors of viral growth could be present in the swabs, such as antibodies or interferons. Interestingly, we found that nasal swabs that were spiked with RVFV could indeed inhibit virus replication by one to two logs, whereas oral swabs had no effect on RVFV replication. Therefore, we would be able to detect infectious particles in nasal swabs containing 10^3^ pfu/mL or higher, but lower levels would be inhibited. This suggests that if infectious virus is present in our nasal swabs, it is below 10^3^ pfu/mL, presumably inhibited and not transmissible. Which component in the nasal swab is inhibitory is not yet known, but it would be interesting to identify it. Despite the lack of infectious virus found in our goat swabs, a few studies have demonstrated horizontal transmission from infectious virus isolated from nasal and oral swabs in sheep [41,55,57]. Hence, further studies in the field with different breeds, species, or viral strains could be useful.

Both the Nubian and LaMancha goats produced low levels of neutralizing antibodies by three dpi, a sharp increase by 14 dpi, and strong peak titers at 21 dpi. These kinetics are similar to what we have seen with the Alpine-Boer goats infected with ZH-501 [36], and are similar to what we have seen in sheep challenged with ZH-501 [35]. Peak levels of neutralizing antibodies were generally similar between the LaMancha and Nubian goats, but were somewhat higher than similar groups in the ZH-501 infected Boer goats [36]. Surprisingly, in contrast to what we have seen previously [35], no difference in neutralizing antibody production was seen between the mammalian and mosquito-derived virus groups in the LaMancha. Based on our previous studies, we hypothesize that the mosquito-derived virus is more efficient at antagonizing interferon responses at the onset of infection, and hence leads to a lower IgG response than the mammalian-derived virus; yet this was not recapitulated in the LaMancha goats. As the LaMancha seemed to be very good at preventing virus infection of the tissues, perhaps this is due to different goat breeds having inherently different susceptibilities and strength of immune responses to RVFV.

## 5. Conclusions

Overall, we have identified a novel goat breed that is useful for RVFV vaccine efficacy testing and have demonstrated that a needle-free intranasal inoculation method produces robust viremia in goats. In addition, we have demonstrated the presence of RVFV RNA in the lymph nodes and the trigeminal nerve at seven dpi after subcutaneous inoculation and many brain tissues remained positive up to 28 days post infection; these tissues could be included as further diagnostic confirmation of RVFV infection in the lab or the field. Importantly, our data also suggests that airborne virus may cause direct invasion of RVFV into the central nervous system rather than through the bloodstream. Finally, a high degree of viral RNA shedding after infection using both subcutaneous and intranasal routes was detected and could be investigated as a non-invasive sampling technique. These findings highlight the impact that a variety of different parameters have on RVFV infection in goats and could be utilized in future vaccine and surveillance studies.
